# Academic neurology in the UK: a plea to turn away from the precipice

**DOI:** 10.1093/brain/awae151

**Published:** 2024-05-21

**Authors:** Helen Devine, Edwin Jabbari, James Scott, Arpan R Mehta, Ruth Dobson, Simon Mead

**Affiliations:** Wellcome Centre for Mitochondrial Research, Clinical and Translational Research Institute, Faculty of Medical Sciences, Newcastle University, Newcastle NE2 4HH, UK; Department of Clinical and Movement Neurosciences, University College London, National Hospital for Neurology and Neurosurgery, London WC1N 3BG, UK; Department of Neurology, Royal Free Hospital, London NW3 2QG, UK; Department of Basic and Clinical Neuroscience, Maurice Wohl Clinical Neuroscience Institute, Institute of Psychiatry, Psychology and Neuroscience, King’s College London, London SE5 9RT, UK; MRC Protein Phosphorylation & Ubiquitylation Unit, School of Life Sciences, University of Dundee, Dundee DD1 5EH, UK; Centre for Preventive Neurology, Wolfson Institute of Population Health, Queen Mary University London, London EC1M 6BQ, UK; Department of Neurology, Royal London Hospital, Barts Health NHS Trust, London E1 1BB, UK; MRC Prion Unit at UCL, Institute of Prion Diseases, University College London, London W1W 7FF, UK

## Abstract

Devine *et al.* argue that recent changes to clinical neurology training in the UK have the potential to exacerbate an existing crisis in academic neurology, and discuss what might be done to remedy the situation.

We should be celebrating. As clinical academic neurologists, the neurological landscape has never looked so promising. The role of clinical academics is to marry clinical experience with basic science research—taking research from the bench through the translational pathway into patient-focused clinical trials. The premise is fantastic: as scientists we experience the joy of curiosity driven work, a degree of autonomy and rare but pivotal moments of scientific discovery; as clinicians we have the privilege of helping patients through deep expertise.

Over recent years, ground-breaking basic science discoveries have led to life-changing improvements in the clinical care we can offer patients in our clinics. From the translational potential of multiomics work untangling the role of CD40L in the pathogenesis of multiple sclerosis,^1^ through use of antisense oligonucleotides which has allowed children with spinal muscular atrophy to walk,^2^ to targeting CGRP receptors in the treatment of migraine^3^ and the first disease modifying treatments for Alzheimer’s disease.^4^ We are in a golden age of neurological innovation.

And yet, we hear academic neurology in the UK is in crisis. Professor Sir Paul Nurse’s independent review of 2023 highlighted the critical need to support clinical academics working in the NHS as a key component of the UK’s research landscape:

‘the Review heard major concerns expressed by clinical researchers that the demands of their clinical training and health care duties were in conflict with their research training, and for the time needed to carry out research. They argued that research activities are being squeezed out and are on a downward trajectory, weakening the ability of the UK to carry out the research needed to make the NHS more effective and efficient, and missing opportunities to boost the economy. The Government needs to tackle this increasingly damaging problem with urgency, to ensure that those clinically trained individuals with the talent to carry out research are able to do so’.^5^

In 2023, the House of Lords Science and Technology Committee conducted an inquiry into the plight of clinical academics and concluded in a letter from Baroness Brown to the Secretary of State that ‘the clinical research environment in the the NHS is on a dangerous precipice and without urgent action we risk losing out on [the benefits of research]’ and the UK’s clinical research capacity could be permanently diminished.^6^

Focusing on neurology academia, 10 years ago, 32% of academic neurologists in the UK were under 35: today that proportion is 16% (Fig. 1).^7,8^ Young neurologists are choosing not, or are unable, to follow an academic career. The number of academic neurologists has fallen in relation to the total number of neurologists on the specialist register, despite an uptick in absolute numbers. Neurology is not alone. The 2023 Clinical academic survey produced by the Medical Schools Council^7^ reveals an ageing workforce with ethnic and gender imbalances across clinical academia.

**Figure 1:**
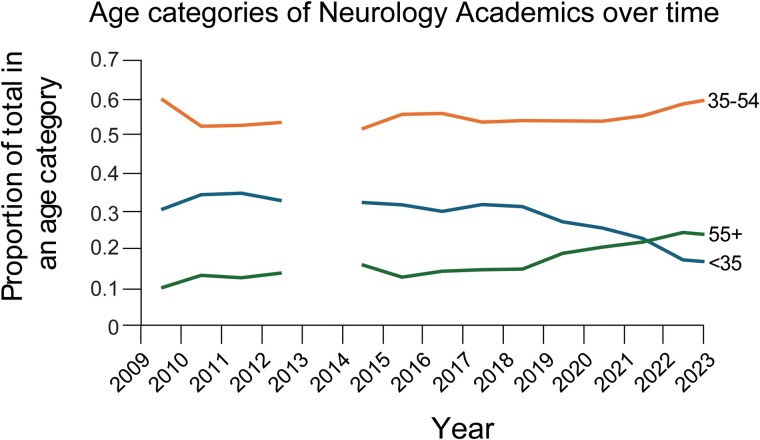
**Changes in the age profiles of academic neurologists over time.** Data were obtained from both the Medical Schools Council^7^ Clinical academic survey and the General Medical Council^8^ Register over time by specialty. Data for 2013 were anomalous and therefore excluded for clarity.

Why is this happening? We are training fewer clinical academics as a proportion of total clinicians, and there are longstanding issues of a leaky pipeline and artificial bottlenecks in training. The steady trickle of loss of talent is becoming a torrent. Major changes in clinical neurology training have recently been introduced in the UK, which will only exacerbate these issues by squeezing the time available to make it as an academic. Here we will describe and discuss these changes and what might be done to remedy the situation.

There are several pathways to the Certificate of Completion of Training (CCT), necessary to work as a consultant academic neurologist in the UK. There is a traditional clinical route composed of three stages (foundation, internal medicine, specialty training) as well as academic equivalents of each stage that incorporate research time known as Integrated Academic Training. Trainees can cross between these two pathways at each stage. The UK Foundation Programme (UKFP) is a 2-year postgraduate training programme in which doctors rotate through a variety of specialties in hospital and community settings every 4  months. The Specialised Foundation Programme (SFP) is an alternative to the UKFP and incorporates research into foundation training, often with a specialty theme. There are around 450 SFP posts in the UK each year. In the past, these have been awarded through a national recruitment process scored according to evidence of academic achievements such as presenting at conferences or publishing in scientific journals. In 2025, the SFP will move to a Preference Informed Allocation system, in line with the UKFP, despite the concerns of senior academic clinicians.^9^ Research achievements will no longer count towards an application, and students will simply rank geographical regions in order of preference and be randomly assigned a ranking and to SFP posts, regardless of whether they have demonstrated any interest in research or the specialty theme of the post. This will surely disincentivize aspirant academics taking intercalated science degrees that are often the first opportunity to catch the research bug.

Following the completion of foundation training, prospective academic neurologists must complete Internal Medicine Training, a 3-year training programme, followed by specialty training in neurology. The Shape of Training reforms to the neurology curriculum, fully introduced in 2022, have the aim of increasing generalism and will undoubtedly have a major impact on both clinical and academic training. In addition to 5 years of specialist neurology training, trainees are required to acquire ‘capabilities in practice’ (CiPs) during internal medicine blocks amounting to 1 year. These are usually taken at the start and end of training, with additional dedicated periods of exposure to the acute medical take throughout training, to obtain a triple CCT. The prospect of attempting to ‘triple train’ in Neurology and Internal Medicine, gain a subspeciality CCT in stroke medicine and on-the-side become a world-class researcher is daunting. The net result of this is that trainees see academic training as infeasible alongside the requirements of clinical training as it risks further long extensions for adequate exposure and competency and triggers concerns around the lack of mastery of core curriculum components (Fig. 2).

**Figure 2:**
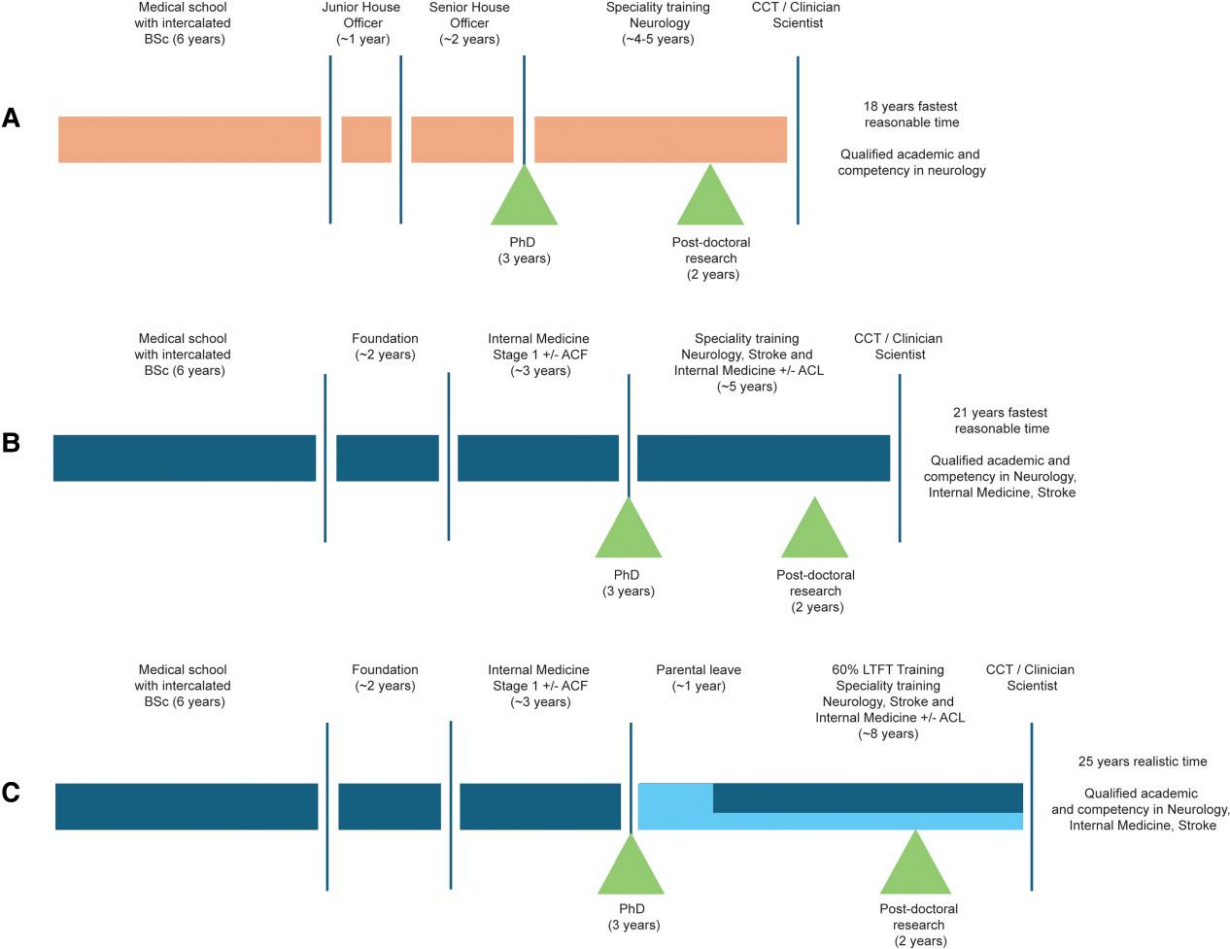
**Flow charts describing possible academic neurology career paths.** These career paths are based on (**A**) the period during which two of the authors trained (R.D. and S.M.) compared with (**B**) those expected for current trainees (J.S. and E.J.). The main changes are the addition to the neurology curriculum of training in internal medicine and stroke medicine as well as additional general and foundation training prior to neurology specialist training. The consequence is a longer period of training overall for a broader set of competencies but with constrained neurology experience. The changes make it much harder to acquire academic skills and progression, whilst still attaining the Certificate of Completion of Training prior to age 40. (**C**) This challenge becomes impossible if one considers the additional impact of parental leave and less than full time training (H.D.).

Financial implications are an unavoidable consequence of delays in obtaining a CCT. Variable approaches to competency versus time-based training means that academic/research time frequently does not count towards progression. Those training less than full time due to care-giving or other reasons require further extensions to training; the cumulative impact of training extensions mean that obtaining a CCT can be delayed substantially for some clinical academics (Fig. 2). The financial implications of these delays are lifelong. This is compounded by the lack of growth in junior doctor salaries and career average pensions on the 2015 Scheme,^10^ meaning that those who spend the longest in training have the lowest career average salaries and therefore lower pensions.

As with academic careers outside of medicine, a leaky pipeline disproportionately impacts female trainees; returning to clinical training following time out for parental leave is challenging, but the additional challenge of regaining academic momentum at this time can be overwhelming, particularly when compounded by childcare or other responsibilities and potentially the inability to travel to take up opportunities. The number of female neurology academics has declined as a proportion from 34% to 29% of all genders over the past 10 years (Fig. 1) when it should be increasing. Ultimately, all of these considerations selectively disadvantage certain groups, including less than full time trainees, those with a caring role and those who do not have the luxury of relying on generational wealth to supplement a loss of earnings. The net result of this is inequitable access to opportunities and a lack of diversity, which is a chronic issue in clinical academia. However, diversity is crucial to the success of clinical academia in terms of research approaches, techniques and the consideration of patient populations.

To widen access to research training and support careers in clinical academic neurology requires urgent and comprehensive interventions at each stage of medical training, and we welcome several aspects of the Government’s response to the House of Lords report. However, there are some specific issues that only we as neurologists can address. The inflexible requirement for generalist training for all neurologists is almost impossible to reconcile with the needs of academic neurology. We propose that committed academic neurology trainees should have the opportunity to opt out of internal medicine dual training and instead use the time to pursue research excellence. For those academics who later decide not, or are unable, to pursue a clinical academic career, post-CCT credentialling in internal medicine could mitigate job concerns. A comprehensive strategy must also alleviate existing bottlenecks through measures such as expanding the number of dedicated intermediate and senior fellowships for clinical academics and pursuing Equality, Diversity and Inclusion (EDI) with mechanisms to make academia more accessible to minority and under-represented groups and the inclusion of centres away from the ‘golden triangle’ within cross-site collaborations. We are disappointed in the withdrawal of specialized calls for clinical academic training posts by some funders and propose that the inclusion of training aspects for early/emerging clinical researchers should be a strong element in making funding decisions on all new academic initiatives in neurology. Even with positive changes to recruiting and retaining academic neurologists, effective mentorship is invaluable in nurturing this group, conceivably facilitated by professional organizations such as the Association of British Neurologists.

Clinical academics have a vital role in the future of the NHS: bringing innovation from basic science, involving patients in clinical trials, supporting the workforce and training the next generation. Research should not be seen as a nice optional extra. It is associated with improved hospital performance and outcomes and ultimately drives improvements in the health and wealth of the nation. However, neurology academia in the UK is in crisis: we are older and less diverse than we should be; there are fewer young clinical academics being trained as a proportion of the workforce; and there is a leaky pipeline with catastrophic loss of talent at key points due to unnecessary and artificial hurdles and penalties. Importantly the neurology speciality needs to reconsider the mandatory training requirement for generalism: our collective strength should be in a diversity of talents and roles.
